# The Effects of Vessel Traffic on the Behavior Patterns of Common Dolphins in the Tagus Estuary (Portugal)

**DOI:** 10.3390/ani14202998

**Published:** 2024-10-17

**Authors:** Iolanda M. Silva, Nádia Jesus, Joana Castro, Ana Rita Luís

**Affiliations:** 1 MARE—Marine and Environmental Sciences Centre/ARNET—Aquatic Research Network/Ispa—Instituto Universitário de Ciências Psicológicas, Sociais e da Vida, 1149-041 Lisbon, Portugal; nadia.a.j.b.m@hotmail.com; 2 AIMM—Associação para a Investigação do Meio Marinho, 1500-399 Lisbon, Portugal; joana.castro@aimmportugal.org; 3 MARE—Marine and Environmental Sciences Centre/ARNET—Aquatic Research Network, Laboratório Marítimo da Guia, Faculdade de Ciências, Universidade de Lisboa, 2750-374 Cascais, Portugal; 4 MARE—Marine and Environmental Sciences Centre/ARNET—Aquatic Research Network, Faculdade de Ciências, Universidade de Lisboa, 1749-016 Lisboa, Portugal; aluis@ispa.pt

**Keywords:** behavioral budgets, cetaceans, common dolphins, *Delphinus delphis*, land-based surveys, Markov chain, Portugal, Tagus estuary, vessel traffic

## Abstract

**Simple Summary:**

This research highlights the effects of vessel traffic on the behavior of common dolphins in the Tagus estuary, Portugal, a densely populated area with heavy commercial and recreational vessel traffic throughout the year. During one year of land-based observations, dolphin sightings were recorded and analyzed. Although common dolphins often displayed neutral reactions to nearby vessels, behavior disruptions were observed, and individuals were more likely to start traveling when vessels were present. This study underlines the potential impact of boat traffic on dolphin behavior, particularly as dolphin sightings and tourism in the area are on the rise. These insights are important to understand potential impact sources.

**Abstract:**

The impact of vessels on dolphin populations has been extensively studied worldwide. The common dolphin, *Delphinus delphis*, has been observed in the Tagus estuary for the past two centuries, and during the last several years, these sightings seem to have increased. This area has high levels of maritime traffic throughout the year, both commercial and recreational. To understand the possible effects of vessel traffic on dolphins’ behavior, land-based observations were carried out from March 2022 to March 2023. For a total of 67 events (48.9 h of dolphin sightings), differences in behavioral budgets were noted. Although “neutral reaction” was the most observed response when vessels were in the vicinity of dolphins, “negative reaction” was also common and five times more abundant than “positive reaction”. The GEE model showed statistical differences between these reaction types (positive, neutral, and negative). Markov chains’ analysis revealed distinct patterns in the behavioral transition probabilities, as dolphins were more likely to switch to a traveling state when vessels were nearby. This study is the first step towards understanding a potential impact source in the area since it is expected that tourism companies expand due to the increase in dolphin sightings in the estuary.

## 1. Introduction

National economies heavily rely on oceans, particularly through international maritime trade, which accounts for 80% of global goods transportation [1]. Due to the increase in maritime traffic worldwide, the need to address the escalating anthropogenic pressures on marine environments has been highlighted in various studies [2,3,4,5,6,7,8]. The distribution of cetaceans seems to be directly affected by vessel presence, with some species exhibiting long-term impacts and an observed increase in mortality rates [9,10]. Many studies suggest that ship-induced disturbance results in a decrease in the energy budget of individuals [2,10,11,12], which influences certain basic functions of life [13], leading to population-level impacts [2,14,15] such as alterations in the use of habitat [16]. Nevertheless, the response levels depend on the species and/or the individuals (i.e., the presence of calves, juveniles, and adults) and the type, number, and movements of the vessels [4,17]. For instance, some species may positively interact with vessels, while others may avoid them, depending on how invasive the interaction becomes [4,7,17].

For species of the Delphinidae family, short-term effects due to marine traffic are usually associated with behavioral changes [2,10], namely modifications in dive behavior [2,18], respiration characteristics [19,20,21], changes in path direction and speed [7,20,22,23], and behavioral budgets [2,10,11,24]. Furthermore, vessel traffic is one of the main causes of underwater noise, which can have serious consequences for cetaceans’ communication [16,25], since underwater sources can affect vocalization rate [24,26,27], and mask bioacoustic signaling [28].

The effects of marine traffic can be cumulative with other known threats such as pollution, habitat loss, and fisheries [29,30], particularly for coastal cetaceans [6,15] whose environment is subject to higher anthropogenic pressure [16].

While it is difficult to understand the long-term implications, behavioral studies can be used to infer about the effects on individuals’ fitness, survival, and, ultimately, their consequences at a population level [31]. Therefore, activity budgets can provide valuable insights into biological responses to a specific threat and can be used as a proxy in impact assessment studies and mitigation strategies [32].

The Tagus estuary is essential for maritime transportation in Portugal, as it serves as the main shipping terminal (Port of Lisbon) of this coastal country [33]. At this location, vessel traffic is very intense, and commercial and recreational vessels are frequent year-round. For centuries, fishing communities have existed along the banks of the Tagus estuary [33], and possible effects of such activities for delphinids include prey depletion, habitat destruction, and bycatch [34]. Additionally, there are several tourism companies dedicated to maritime activities, including five dolphin-watching companies [33].

One of the several species found in the Tagus estuary is the common dolphin, *Delphinus delphis*. Common dolphins are known for their social behavior [35], and the literature documents the frequent occurrence of common dolphins approaching vessels [2,36], which makes them susceptible to potential cumulative effects [2,6].

Given the charismatic nature of cetaceans, the dolphin-watching industry is expected to develop wherever the existence of these species is known [37], and such activities may have a significant impact on dolphins’ behavior.

This study provides the first insight into the interactions between common dolphins and vessels in the Tagus estuary, Portugal, with baseline information on the behavioral effects of vessel traffic in the region. Here, the effects of vessels’ presence on the dolphins’ behavioral budget and behavioral transitions in the Tagus estuary were investigated and studied.

## 2. Materials and Methods

### 2.1. Study Area

**Figure 1:**
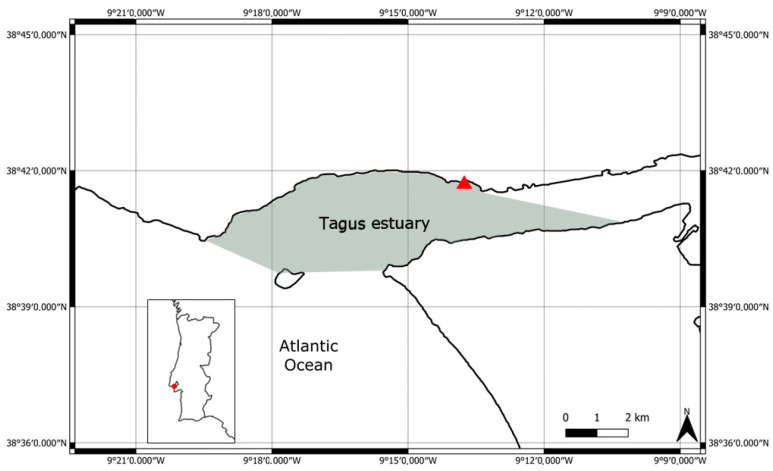
Map of the study area. The red triangle represents the Vessel Traffic Services (VTS) tower where the land-based observations were conducted.

The study area is located at the lower section of the Tagus estuary (GPS coordinates for the center of the area are 38°40′56.3″, 9°15′55.7″) and covers an area of approximately 28 km^2^. The Tagus estuary is a mesotidal estuary with semidiurnal tides located on the central west coast of Portugal [38]. The lower section of the estuary is linked to the Atlantic Ocean through an extended, narrow inlet [38] of sand and silt, with depths ranging from 25 to 49 m [38,39] (Figure 1).

Common vessels (such as cargo ships, tankers, and others) are common throughout the year due to the navigation channel that serves the busiest shipping terminal of Portugal—Port of Lisbon [40]. Other types of vessels present include passenger transportation vessels, such as ferry boats, fishing, recreational, and tourism vessels [33,39].

### 2.2. Data Collection

Land-based observations were conducted from a high vantage point on the top floor of the VTSs (Vessel Traffic Services) tower in Algés, Lisbon, which reached a height of 38 m. From March 2022 to March 2023, a team with a minimum of two trained observers undertook shifts between 08:00 and 16:00 every other day. These surveys were conducted on days with high visibility (up to a radius of 5 km), sea state conditions of Beaufort < 4, and absence of precipitation.

To detect the presence of dolphin groups, the study area was scanned by the observers every 5 min, with binoculars (Cannon 12 × 36 IS III and Cannon 10 × 30 IS II) and a telescope (Nikon PROSTAFF 5 82 mm with 20–60 × magnification), sequentially. Scans were conducted from left to right, covering the entire study area. As soon as cetaceans were sighted, continuous group sampling started, and information regarding species, behavior patterns, and presence/absence of vessels was recorded in 5 min sampling periods until the group was lost or left the study area. For this study, a “group” was defined as an association of relatively close (±30 m) dolphins that exhibited a common pattern of behavior [24]. Behavior patterns were categorized as *feeding activities*, *traveling*, *socializing,* and *resting* (Table 1).

The most frequent behavioral pattern observed during the 5 min sample was defined as the dominant activity state. Vessels were considered near the focal group if they were within a radius of approximately 200 m, and dolphins’ reactions were classified as (i) positive if dolphins actively approached the vessels and/or bowriding was observed, (ii) neutral if dolphins maintained the behavioral pattern and/or, in case of traveling, maintained the previous direction, or (iii) negative if dolphins changed/stopped the behavioral pattern, changed group formation, increased diving periods, and/or displayed tailslaps.

### 2.3. Video and Audio Recording Analysis

For complementary information regarding the dolphins’ behavior, sightings were recorded with a Canon Legria HF R606 camera. Video and audio samples were analyzed through the VLC Media Player software (version 3.0.17.3), and all information regarding the location, group composition, and behavior was documented. Subsequently, this information was cross-checked with on-site records.

### 2.4. Statistical Analysis

For statistical analysis, continuous recordings were grouped in “events”. Each event included all 5 min blocks from the first sighting until the group was lost or left the area. A 15 min interval in between events was established to assure sample independence.

To assess the activity behavior budgets and dolphins’ reactions to vessels, *socializing* was excluded from statistical analysis due to the limited number of observations (N = 11, respectively).

To study the effect of vessels on the behavior of common dolphins, activity budgets were calculated according to the following scenarios:

1. Absence of vessels;
2. Presence of vessels.

For each event, time spent on each behavior category (*feeding activity* and *traveling*) was summed for the two previous scenarios. A Shapiro–Wilk test and a Levene’s test were applied to test the normality and homogeneity of variance, respectively. Since data did not present normality and homoscedasticity, even when outliers were removed, we used all available data for statistical analysis. The Mann–Whitney U test was used to compare the activity behavioral budgets of common dolphins in the presence and absence of vessels.

To further understand the possible effects of vessels on dolphins’ behavior, the reactions of dolphins to vessels were analyzed using generalized estimating equations (GEEs). When the data are in clusters, this statistical test can evaluate the correlation within a cluster, assuming the independence in the different clusters [42]. The baseline category for the behavioral pattern was *traveling*, while *neutral* was defined for dolphins’ reactions to vessels. Using the RStudio package ‘geepack’ [42] with an exchangeable correlation structure and a gamma distribution, three models were developed: (1) the time spent as a function of the reaction to vessels; (2) the time spent as a function of the reaction to vessels and the different behavior patterns; and (3) the time spent as a function of the reaction to vessels, the different behavior patterns, and the relationship between these two. According to Halekoh and colleagues [42], the exchangeable correlation structure is highly recommended when the data have categorical variables. To assess the model that best fits the data, the ANOVA method was used, comparing the models using the Wald test statistic [42].

Finally, Markov chains were used to assess dolphins’ behavioral transitions in two possible scenarios: (1) control scenario, with no vessels; (2) impact scenario, with vessels in proximity to the dolphins. Based on Lusseau [31], the transition probabilities for the two scenarios were calculated using the following equation:

$$p_{ij}=\frac{a_{ij}}{\sum_{j=1}^{5}a_{ij}},\;\sum_{j=1}^{5}p_{ij}=1,$$

where *a_ij_* is the number of transitions observed from *i* to *j*, and *p_ij_* is the transition probability from *i* to *j*. All behavior patterns were used in this analysis, and the RStudio package utilized was ‘markovchain’.

All statistical analyses were conducted in RStudio version 4.2.3.

## 3. Results

During a total of 942.9 h (in 147 days) of land-based observations, common dolphins were sighted for 48.9 h (in 38 days), accounting for 5% of the total sampling time. Dolphin sightings corresponded to 67 events and a total of 575 5 min behavioral samples (56% without vessels and 43% in the presence of vessels).

**Figure 2:**
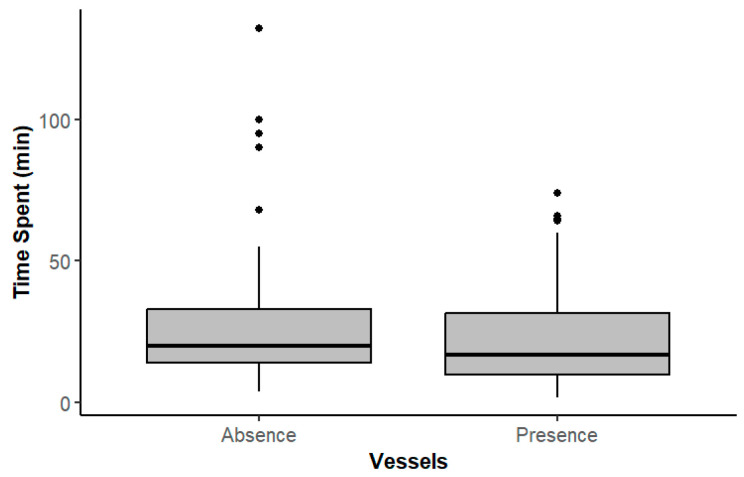
Time spent, in minutes, in the study area when dolphins were observed in the absence or presence of vessels.

In general, dolphins spent an average of 28.3 ± 3.46 min daily in the study area when there were no vessels in their vicinity. In this scenario, the minimum time of observation was 4 min, and the maximum was 132 min. The average time dolphins spent with vessels was slightly less, 23.5 ± 2.55 min, ranging between 2 and 74 min (Figure 2). These differences were not statistically significant (U = 3124, *p =* 0.7).

### 3.1. Activity Behavioral Budgets

Overall, common dolphins spent most of their time *traveling* (60.5%) and in *feeding activities* (35.8%). *Socializing* comprises 1.9% of common dolphins’ activity budget in the lower section of the Tagus estuary. *Resting* activity was never recorded in this study.

**Figure 3:**
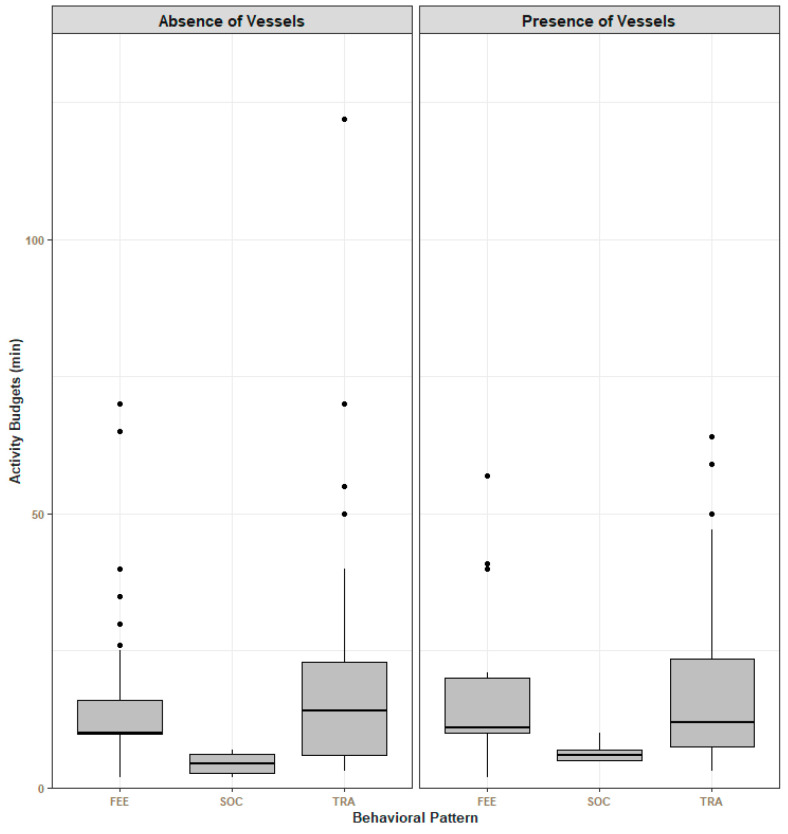
Average time that dolphins spent in each behavior in the absence (dark gray) or in the presence (light gray) of vessels. The average time was obtained through the sum of the time spent in each behavioral state within each event. Error bars represent the standard error. FEE—feeding activities; SOC—socializing; TRA—traveling.

When vessels were within a 200 m radius, dolphins spent less time *traveling* (N _absence of vessels_ = 179, 18.7 min vs. N _presence of vessels_ = 143, 17.4 min), while the time spent *socializing* increased (N _absence of vessels_ = 4, 4.5 min vs. N _presence of vessels_ = 6, 6.6 min). The time spent in *feeding activities* was similar in the two scenarios (N _absence of vessels_ = 128, 16.30 min vs. N _presence of vessels_ = 78, 16.37 min). When comparing the behavioral states of *feeding activities* and *traveling*, no significant differences were found between the two scenarios (feeding activities: U = 522, *p* = 0.8; traveling: U = 1113, *p* = 0.8) (Figure 3).

### 3.2. Reaction to Vessels

In the presence of vessels, dolphins exhibited, mostly, a neutral reaction (80.2% of the observation time). Although other reaction categories were less frequent, negative responses (15.9%) were nearly five times as numerous as the positive ones (3.9%).

**Figure 4:**
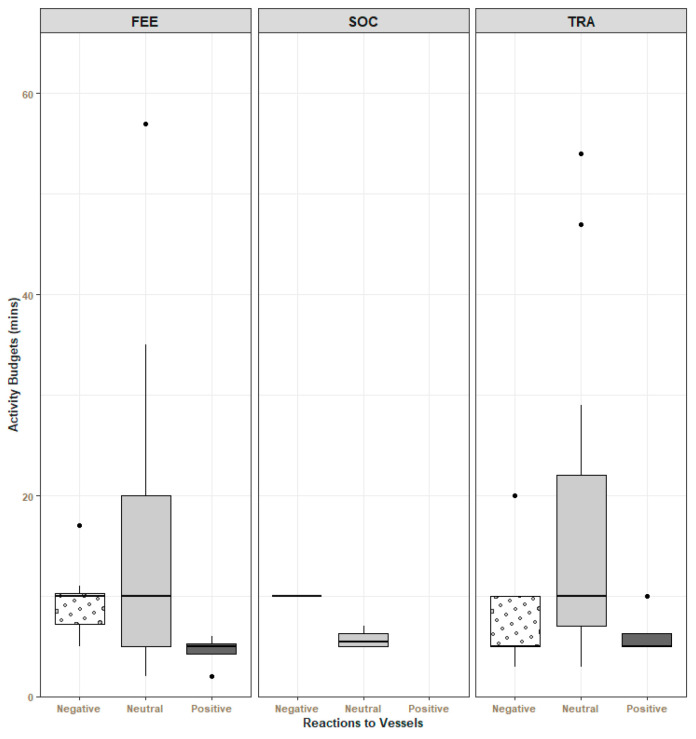
Reaction of dolphins to the vessels during each behavior observed. The average time that dolphins spent in the different reactions was obtained through the sum of the time spent in each behavioral state and reaction within each event. Error bars represent the standard error. FEE—feeding activities; SOC—socializing; TRA—traveling.

During *traveling* and *feeding activities*, “neutral” reaction was the most common response to vessels (15.2 ± 1.77 min, 13.5 ± 2.4 min, respectively), followed by “negative” (7.53 ± 1.09 min, 9.5 ± 1.35 min, respectively) and “positive” (6.25 ± 1.25 min, 4.5 ± 0.87 min, respectively). When dolphins were *socializing*, only negative and neutral reactions were recorded (10 and 5.75 ± 0.48 min, respectively) (Figure 4).

**Figure 5:**
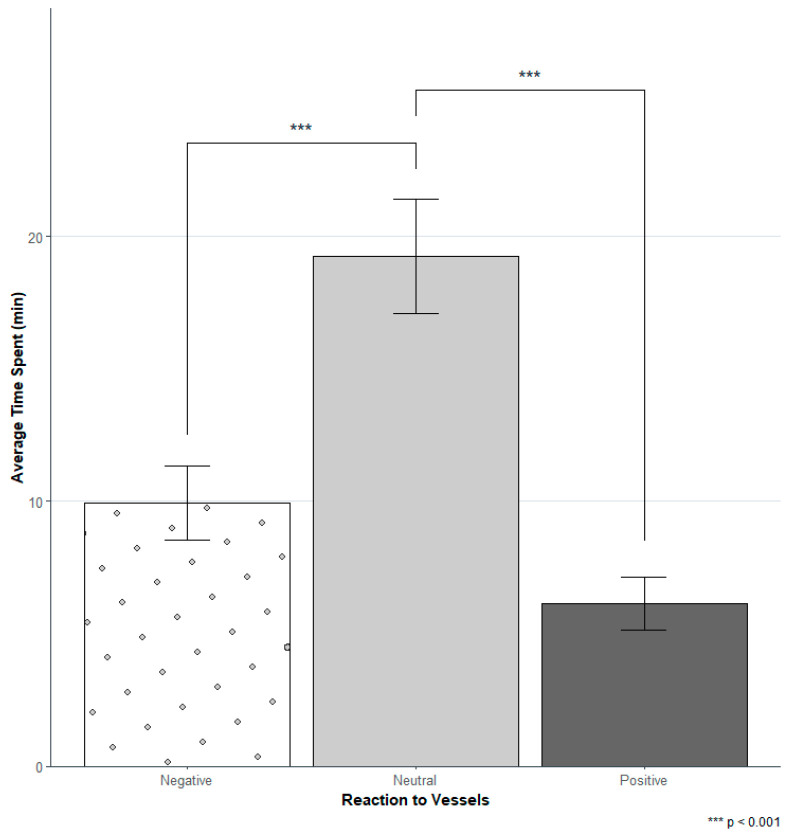
Average time spent in each reaction to vessels. The average time that dolphins spent in the different reactions was obtained through the sum of the time spent in each behavioral state and reaction within each event. The statistical results are provided from the GEE.

Overall, the time that dolphins spent in negative and positive reactions was significantly less than the time they spent exhibiting neutral reactions (negative: Wald χ^2^ (1) = 14.82, ρ = 0.00012; positive: Wald χ^2^ (1) = 18.07, ρ = 2.1 × 10^−5^) (Figure 5).

Using the GEE statistical approach, there were three potential models that could further explain the possible effects of vessels on dolphin behavior. Wald’s test revealed the third model (time spent as a function of the reaction to vessels, the different behavior patterns, and the relationship between these two) as the best fit (*p* = 0.14) (Table 2), with a weak negative correlation between observations within an event (α = −0.0582).

### 3.3. Behavioral Transitions

**Figure 6:**
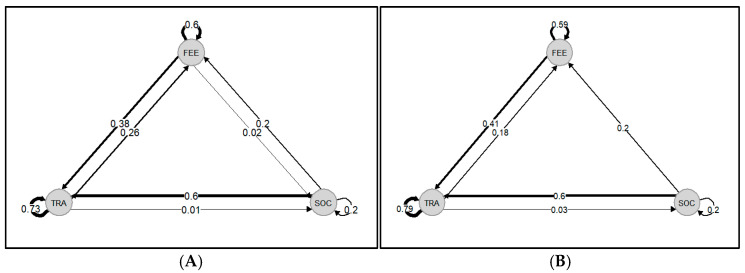
Markov chains representing behavioral transition probabilities: (**A**) control chain; (**B**) impact chain. FEE—feeding activities; SOC—socializing; TRA—traveling.

When dolphins were sighted without vessels in their vicinity, the transitions that occurred more often were *traveling* to *traveling* (P_TRA-TRA_ = 0.73), *socializing* to *traveling* (P_SOC-TRA_ = 0.6), and *feeding activities* to *feeding activities* (P_FEE-FEE_ = 0.6). Similarly, the most common transitions when vessels were in proximity to the dolphins were *traveling* to *traveling* (P_TRA-TRA_ = 0.79), *socializing* to *traveling* (P_SOC-TRA_ = 0.6), and *feeding activities* to *feeding activities* (P_FEE-FEE_ = 0.59). Nonetheless, the transition *feeding activities* to *traveling* slightly increased (P_FEE-TRA_ = 0.41) when vessels were in the vicinity of dolphins. Opposite, the transition *traveling* to *feeding activities* showed a decrease in the scenario with vessels’ presence (P_TRA-FOR_ = 0.18). Moreover, in the presence of vessels, the transition from *feeding activities* to *socializing* was not observed (Figure 6).

## 4. Discussion

Assessing the effects of vessels on cetaceans always poses a challenge, especially when there is no baseline information on species occurrence and habitat use for a specific site.

Land-based observations, as a non-invasive behavioral observation tool, provide information that may aid in setting referential status and, simultaneously, infer possible impacts of anthropogenic activities.

This first study on common dolphin behavior in the Tagus estuary, Portugal, illustrates the influence of vessels on dolphins’ daily activities. At this site, common dolphins were observed regularly, year-round, and three of the four behavioral states were recorded, with *traveling* and *feeding* as the dominant activities. These observations provide a valuable insight into the habitat use of common dolphins in estuarine environments, especially because sightings of common dolphins are rare in these habitats. In other regions, environmental factors, such as salinity and temperature, are known to influence the presence of dolphins nearshore [43,44,45]. Additionally, the presence of vessels has been documented as a possible constraint to delphinids occurring in coastal areas [11,15,24]. In this study, the time that dolphins spent in the lower section of the Tagus estuary with and without vessels was very similar (Figure 2), which possibly indicates that the presence of vessels does not limit the time spent in the area.

Other dolphin species are known to occur in estuaries, even with residency patterns, which suggest that the use of such habitats can be mostly determined by other factors, such as prey availability [33,46,47,48,49,50]. According to Cremer & Simões-Lopes [51], the occurrence of franciscana dolphins (*Pontoporia blainvillei)* in Babitonga Bay is directly linked to food availability. Still, researchers have specified vessel traffic as the second limiting factor for dolphins’ occurrence in these environments, and some populations are known to experience short-term effects due to frequent vessel presence [46,47,48]. Since common dolphins’ occurrence in estuaries is rare, the importance of these nutrient-rich areas for local populations and the consequences of vessels’ presence are yet to be assessed.

In this study, dolphins were observed in *feeding activities*, *traveling,* and *socializing* in both conditions (with and without vessels). Although only slight differences were found in the behavioral budgets, it is interesting to note that when vessels were present, dolphins spent less time *traveling*, while the time spent in *feeding activities* and *socializing* increased (Figure 3). In a study by Christiansen and colleagues [52], bottlenose dolphins were recorded increasing foraging behavior during interactions with tour boats, which was discussed as a possible strategy to compensate for energy lost during these encounters. Furthermore, Marley and colleagues [24] explained that prey behaviors may change in the presence of vessels, leading to easier predation by the dolphins. Other studies reported that when dolphins are disturbed by vessels, they exhibit vertical avoidance, and surface behavior can be easily misidentified [24,31]. In a study by Ng & Leung [53], dolphins were observed to have longer dives when vessel traffic was very intense. Thus, it is possible that the increase in *feeding activities* when vessels were present might be a result of misclassification of evasive maneuvers due to the observation of scattered groups and longer submersions. Both hypotheses are valid scenarios for the Tagus estuary, and continuous monitoring of common dolphins’ occurrence, combined with bioacoustics data, could help to explain the differences in behavioral budgets.

To clarify the effects on dolphins’ behavior, an analysis was conducted on the reactions of dolphins during the encounters with vessels. In most of the events, dolphins exhibited a neutral reaction to vessels’ presence (Figure 4). The neutral reaction might indicate the absence of immediate disturbance, as it has been previously reported for other delphinids, when assessing behavioral responses to cargo ships [53], one of the most common vessel types in the Tagus estuary. It is important to note that certain variables or circumstances may have the potential to change the outcome of the scenario in question. The response to vessels can be influenced according to vessel type, exposure duration, and number of vessels [9,24,31]. The study by Marley and colleagues [24] reports changes in dolphins’ behavioral budgets according to the number of vessels. Furthermore, according to a study by Papale and colleagues [54], negative reactions occurred in 70% of the encounters between dolphins and speedboats, resulting in changes to their behavior. The unpredictability of fast-moving watercraft seems to impact dolphins’ responses [53]. Additionally, many studies have shown the impact of underwater noise on cetaceans [24,25,26,55]. In this study, it was not possible to explore all of these factors due to the limited number of samples for the different conditions. Still, it was possible to verify distinct responses to vessels according to the dolphins’ behavior. Negative reactions were observed during *feeding activities*, *socializing,* and *traveling*, indicating that these behaviors could have been affected by the vessels’ presence at some point.

Evaluating these short-term impacts may help to assess long-term effects. Previous research consistently shows that individuals frequently alter their behavior in response to approaching vessels as a direct method of avoidance [56]. Our findings regarding behavioral transitions are in line with these results (Figure 6). The transitions from *feeding activities* to *traveling* increased when vessels were present. This might be a clear indicator of behavioral disruption as a direct reaction to a vessel interaction [56]. If feeding behavior is constantly disrupted, it could have serious consequences for the individuals’ fitness and effects at the population level. It has been found in previous research that reducing food intake can result in lasting consequences on an individual’s reproductive success and overall physical health and trigger nutritional distress [6,56,57]. Although the literature does not provide specific details about the potential impact on nursing groups, research has demonstrated that pregnant and lactating females adjust their diet to optimize their energy intake and meet their nutritional needs [6,58,59]. Groups of common dolphins sighted in the Tagus estuary, Portugal, often include calves and juveniles who may be more vulnerable to vessel interactions. Furthermore, in the presence of vessels, the transition from *feeding activities* to *socializing* ceased to occur, which could also signal behavioral disruption with long-term effects.

## 5. Conclusions

Sightings of common dolphins in estuaries are very unusual, but in the Tagus estuary, the presence of groups with calves and juveniles is now regular [33]. The presence of young individuals may influence the observed behavior and possibly the reaction to vessels; thus, it is important to understand how the encounters with vessels may influence the dynamic of mother–calf dyads. In this study, it was possible to observe small differences in behavioral budgets when vessels were near dolphins, negative reactions to vessels, and changes in behavioral transitions during *feeding activities*. It is essential to look at these results closely, especially considering that *feeding activities* were the second-most recorded behavior and that the Tagus estuary might be an important feeding area for these dolphins.

Furthermore, maritime traffic in this area is increasing, and dolphin-watching companies could expand their activities due to the increase in sightings. For these reasons, it is critical to assess the factors that might influence dolphins’ responses to vessels in future studies.

## Figures and Tables

**Table 1:** Descriptions of the behavioral patterns considered in this study, adapted from Neumann [41].

Behavioral Pattern (Abbreviation)	Definition
Feeding activities (FEE)	Individuals change direction frequently with variable diving periods (medium submersions (±1 min) to longer submersions (>1 min)).
Traveling (TRA)	Individuals move in a consistent direction with variable diving periods.
Socializing (SOC)	Individuals move in different directions with short submersions. It is frequent to find dolphins in physical contact with one another and having synchronized movements.
Resting (RES)	Individuals clustered at the surface in a constant direction.

**Table 2:** Comparison of the models by the Wald test. The model formula is structured as “response variable ~predictor variable”, with the operator ~ meaning “as function of” and * meaning “interaction between covariates”. DF—degrees of freedom.

Candidate Models		
M1: Time~reaction to vessels M2: Time~reaction to vessels + activity patterns M3: Time~reaction to vessels + activity patterns + reaction to vessels * activity patterns		
**Model comparison**	**DF**	***p*** **-Value**
M1 vs. M2	1	0.75
M1 vs. M3	3	0.27
M3 vs. M3	2	0.14

## Data Availability

The raw data supporting the conclusions of this article will be made available by the authors on request.
